# Supplementary material for: Tead1a Initiates Transcriptional Priming Through the TEAD1a/YAP‐Notch1‐Spi1/Cebpα Axis to Promote Neutrophil Fate
Source: Adv Sci (Weinh). 2025 Aug 28;12(41):e05441. doi: 10.1002/advs.202505441 (PMC12591127; doi:10.1002/advs.202505441)
Supplement: Supplementary file 4 — Supplementary Dataset [file ADVS-12-e05441-s004.zip › 3-6.zip!/3-6/02-6.cluster/cluster16/cluster16_vlnplot.pdf]

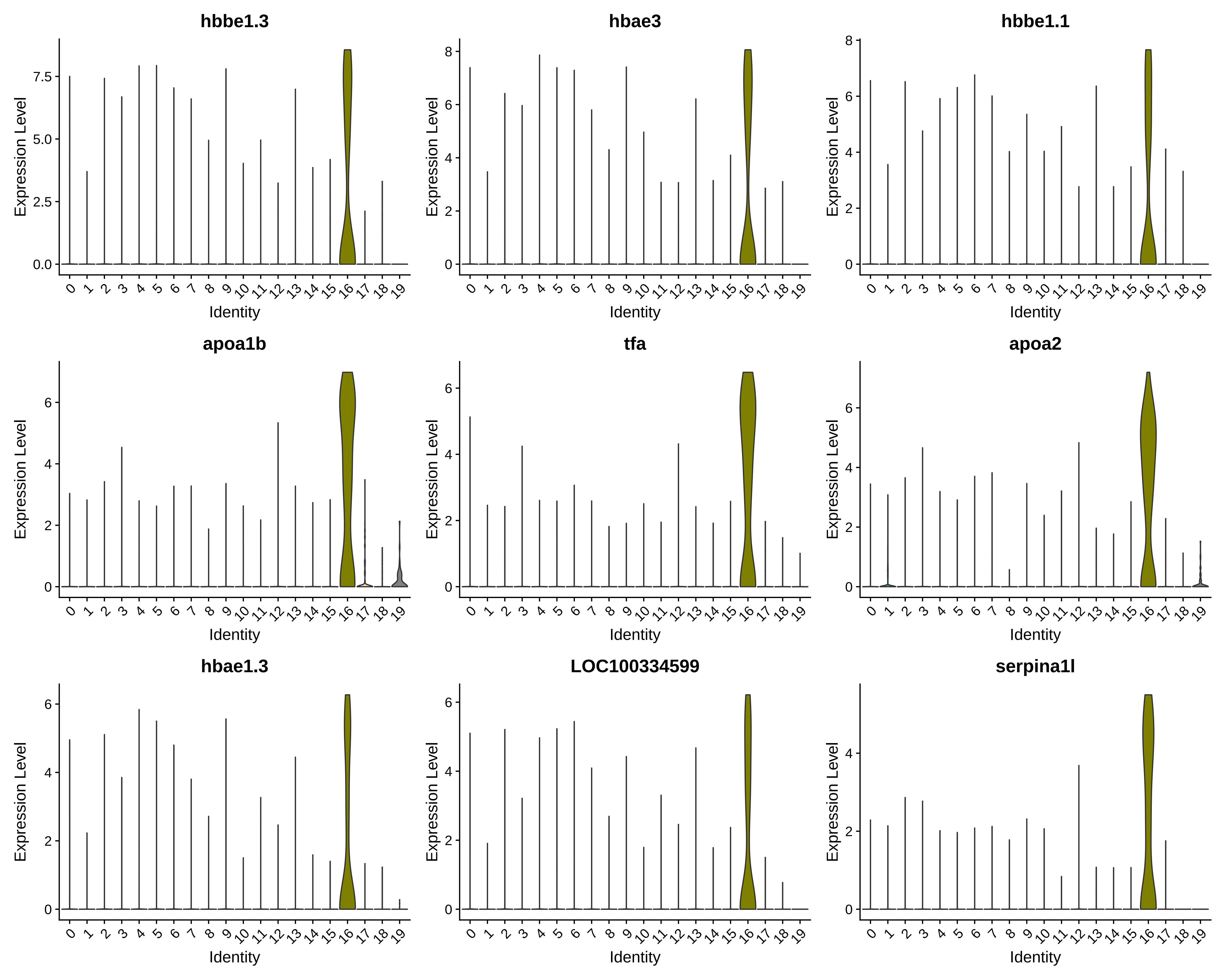
hbbe1.3
hbae3
hbbe1.1
apoa1b
tfa
apoa2
hbae1.3
LOC100334599
serpina1l
Expression Level
Identity
0 1 2 3 4 5 6 7 8 9 10 11 12 13 14 15 16 17 18 19
0.0
2.5
5.0
7.5
0
2
4
6
8
